# Parasympathetic Activity and Blood Catecholamine Responses Following a Single Partial-Body Cryostimulation and a Whole-Body Cryostimulation

**DOI:** 10.1371/journal.pone.0072658

**Published:** 2013-08-22

**Authors:** Christophe Hausswirth, Karine Schaal, Yann Le Meur, François Bieuzen, Jean-Robert Filliard, Marielle Volondat, Julien Louis

**Affiliations:** 1 Research department, Sport Expertise and Performance (SEP) Laboratory, National Institute of Sport, Expertise and Performance (INSEP), Paris, France; 2 Sports Performance Laboratory, Sports Medicine Program, University of California Davis, Sacramento, California, United States of America; 3 Medical department, National Institute of Sport, Expertise and Performance (INSEP), Paris, France; Universidad Europea de Madrid, Spain

## Abstract

The aim of this study was to compare the effects of a single whole-body cryostimulation (WBC) and a partial-body cryostimulation (PBC) (i.e., not exposing the head to cold) on indices of parasympathetic activity and blood catecholamines. Two groups of 15 participants were assigned either to a 3-min WBC or PBC session, while 10 participants constituted a control group (CON) not receiving any cryostimulation. Changes in thermal, physiological and subjective variables were recorded before and during the 20-min after each cryostimulation. According to a qualitative statistical analysis, an *almost certain* decrease in skin temperature was reported for all body regions immediately after the WBC (mean decrease±90% CL, -13.7±0.7°C) and PBC (-8.3±0.3°C), which persisted up to 20-min after the session. The tympanic temperature *almost certainly* decreased only after the WBC session (-0.32±0.04°C). Systolic and diastolic blood pressures were *very likely* increased after the WBC session, whereas these changes were trivial in the other groups. In addition, heart rate *almost certainly* decreased after PBC (-10.9%) and WBC (-15.2%) sessions, in a *likely* greater proportion for WBC compared to PBC. Resting vagal-related heart rate variability indices (the root-mean square difference of successive normal R-R intervals, RMSSD, and high frequency band, HF) were *very likely* increased after PBC (RMSSD: +54.4%, HF: +138%) and WBC (RMSSD: +85.2%, HF: +632%) sessions without any marked difference between groups. Plasma norepinephrine concentrations were *likely* to *very likely* increased after PBC (+57.4%) and WBC (+76.2%), respectively. Finally, cold and comfort sensations were *almost certainly* altered after WBC and PBC, sensation of discomfort being *likely* more pronounced after WBC than PBC. Both acute cryostimulation techniques effectively stimulated the autonomic nervous system (ANS), with a predominance of parasympathetic tone activation. The results of this study also suggest that a whole-body cold exposure induced a larger stimulation of the ANS compared to partial-body cold exposure.

## Introduction

The first very low temperature cold rooms, a peculiar form of cryostimulation appeared in Japan in 1981, when Yamauchi successfully used a cryogenic chamber to treat rheumatism [1]. Whole-body cryotherapy (WBC), as it is known today, consists of acute exposure to very cold air in special cryochambers. The air is maintained at temperatures ranging from -110 to -160°C, limiting exposure to 3-4 minutes [2]. One of the most well-established physiological responses to cold exposure is triggered by the decrease in skin temperature, promptly stimulating cutaneous receptors and their sensory afferents to excite sympathetic adrenergic fibers, in turn causing the constriction of local arterioles and venules. The resulting decrease in blood flow to the periphery or injured/inflamed tissues, reduces local metabolic processes, thereby attenuating the inflammatory response and the formation of oedema around the injured tissues [3]. It has been shown that cryotherapy reduces cell necrosis and neutrophil migration and slows cell metabolism and nerve conduction velocity, which in turn reduce secondary tissue damage and pain sensation [4].

The medical indications for WBC were subsequently extended to various inflammatory conditions – arthritis and multiple sclerosis [5–7], rheumatoid arthritis [8] – and to skin disorders such as psoriasis [5]. The reported reasons for using WBC include decreased joint pain and disorders, improved general well-being, decreased fatigue perception [9] and reduced symptoms of psychiatric disorders such as anxiety and depression [10]. WBC is also extensively used in self-treatment or body hardening against respiratory tract infections and musculoskeletal pain [11], as well as parasympathetic reactivation after intensive exercise [12].

In the sporting realm, WBC (in this instance, more accurately defined as whole-body cryostimulation) has been used at temperatures ranging from -110 °C to -160 °C with the aim of limiting the spread of muscle lesions after training or competing [13]. It has also been offered as a prophylactic treatment to reduce the risk of muscle lesions during intense training periods and to increase the antioxydant status after multiple exposures [14]. Despite the increasing popularity of WBC in sports, only few studies have assessed its efficiency in accelerating the recovery of the athlete [9,15,16]. Very recently, post-exercise cold water immersion has been shown to aid recovery by altering blood flow [17], and improving perceptions of recovery [18] which may be reflected by changes in cardiac autonomic activity [19]. WBC may also exert important effects on post-exercise recovery at the cardiovascular level. As exercise causes an intensity-dependent parasympathetic withdrawal and sympathetic increase, a prompt recovery of parasympathetic activity is desirable after exercise. Changes in cardiac parasympathetic activity as assessed by heart rate variability (HRV) analysis have emerged in the literature as a global recovery index that reflects the acute response of the body to exercise; an elevated level of parasympathetic activity allowing rapid cardiodeceleration and faster recovery [18,20,21]. More generally, outside of a sporting context, an elevated parasympathetic activity would also confer a cardioprotective background, dramatically limiting the risk of mortality, and even the occurrence of sudden death [22,23]. While Stanley et al. [18] demonstrated that both cold water immersion (CWI, 5-min in 14°C water) and contrast water therapy (CWT), consisting of 3 cycles alternating immersion in cold (1-min, 14.2°C) and warm (2-min, 35.5°C) water, significantly aided post-exercise parasympathetic reactivation compared to passive recovery in trained endurance athletes, they also reported that this effect was larger with CWI than CWT, suggesting that combining a greater cold stimulus increased the effectiveness of water immersion. While various water immersion protocols have been shown to accelerate post-exercise parasympathetic reactivation, the effect of dry air whole-body cryostimulation (WBC, range from -110°C to -160°C) on post-exercise autonomic recovery is not well documented, even though this recovery method has become increasingly used in high level sport [9,15]. Only one study reported a significant increase in the heat rate variability (HRV) indices of parasympathetic activity following a WBC session performed after exercise in elite synchronized swimmers [12]. Similarly, in resting conditions, Westerlund et al. [24] found that a single session of WBC significantly augmented HRV indices of parasympathetic modulation in healthy nonathletic women, with a mean increase of approximately 50% in RMSSD and high frequency power. Cold exposure has been found to suppress cardiac sympathetic activity and increase parasympathetic output as a result of arterial baroreflex activation [25]. Cold stimulation triggers peripheral vasoconstriction, leading to a shift in blood volume toward the core [26]. The resulting increase in central pressure in turn activates the baroreflex, responsible for reducing sympathetic nerve activity while shifting autonomic heart rate control toward a parasympathetic dominance. However, in the case of healthy recreationally active men training only a few times per month, the autonomic response to WBC has not been investigated. Further, the influence of WBC on blood parameters and subsequent cardiac and thermal responses compared to partial-body cryostimulation (PBC) technique has not been evaluated.

Modern cryotherapy techniques involve local, partial-body and whole-body exposures. WBC and PBC have been developed very recently and many devices are commercially available. The major differences in the two systems are 1) the temperatures (-110°C *vs.* -160°C, for WBC and PBC, respectively), 2) whether the head is exposed to the cold stimulus (yes *vs.* no, for WBC and PBC, respectively), 3) the source of cold stimulation (compressor *vs.* nitrogen gas, for WBC and PBC, respectively). The infrared studies of the temperature response to 3 minutes WBC exposure reported that cold air on the entire human body is responsible of an obvious drop off in skin temperature whereas central temperature did not exceed the thermoregulation range during cryotherapy sessions [27]. Thermal mapping of the body could be influenced by local blood flow, degenerative and inflammatory state of the tissue. It was therefore previously reported that an enhancement of skin temperature profile could increase the diagnostic sensitivity of infrared imaging in patients [28]. The use of several types of cryostimulation raises new questions such as; ‘what is the optimal modality?, What are the duration and minimal temperature required to elicit physiological responses? Is head exposure needed to induce general modifications?’. During WBC the entire body is exposed to cold, including the face and neck, as opposed to a PBC session. It has been shown that the direct effect of cold on the head alone, via face immersion in cold water (without breath holding) aided parasympathetic reactivation significantly following exercise [20]. This increase in vagal tone in response to cold stimuli applied to the face is thought to be principally mediated by trigeminal brain stem pathways rather than by the baroreflex [29]. Further, Eckberg et al. [30] showed that stimulating trigeminal cutaneous receptors by cold water face immersion augmented the magnitude of the vagal response induced by baroreflex activation alone.

The aim of this study was to compare the magnitude of the effects of a single session of WBC *versus* PBC on skin and tympanic temperatures, cold and comfort sensations, and the modulation of the autonomic nervous system (ANS). We tested the hypothesis that WBC would induce a larger increase in plasma catecholamines, and a larger increase in the parasympathetic modulation of heart rate compared to PBC.

## Materials and Methods

### Ethical standards

These experiments were conducted according to the Declaration of Helsinki (1964: revised in 2001) and the protocol was approved by the local Ethics Committee (Ile-de-France, Paris, France) before its initiation. Participants were volunteers and were informed about the study protocol, the risks of all tests, their rights, and they gave their written informed consent before the initiation of the experiment. Further, according to the policies of PLoS Journals for the respect of participant privacy and anonymity, the person figuring in the infrared thermal images of the article has given his written informed consent to publication of his image in a PLoS Journal.

### Participants

Forty healthy men volunteered to participate in this study (see Table 1 for characteristics). Before the experiment a physician examined all the participants to ensure that they did not present any contraindications to intense cold exposure such as cold hypersensitivity (Raynaud’s syndrome), history of heart disease or circulatory pathologies. All participants were recreational athletes between 20 and 55 years of age and were not accustomed to cryotherapy treatments. Since body fat mass can influence the physiological response to cryostimulation [31], body composition was controlled and measured using an 8 point bio-impedance device (InBody 720; 1-1000 kHz, Biospace company, Ltd., Seoul, Korea) validated for accuracy and repeatability [32,33]. All subjects were requested not to smoke, and not to drink any alcohol or hot drinks for 4 h prior to each cold exposure in order to avoid influencing the recorded variables. In addition, subjects were required not to undertake exercise for 24-h prior to each laboratory session.

**Table 1:** Characteristics of participants composing the three experimental groups; CON, Control; PBC, Partial-body cryostimulation, and WBC, Whole-body cryostimulation.

	**CON**	**PBC**	**WBC**
N	10	15	15
Age (year)	33.9 ± 12.3	34.6 ± 11.5	33.3 ± 13.8
Height (m)	1.77 ± 0.06	1.78 ± 0.07	1.77 ± 0.05
Body mass (kg)	74.4 ± 11.8	76.7 ± 8.6	78.5 ± 10.6
BMI (kg. m^-2^)	23.6 ± 3.1	24.2 ± 3.0	25.0 ± 3.3
Fat mass (%)	13.3 ± 6.0	14.3 ± 5.8	14.6 ± 5.4

Values are means ± standard deviations

BMI, Body Mass Index

### Study design

**Figure 1:**
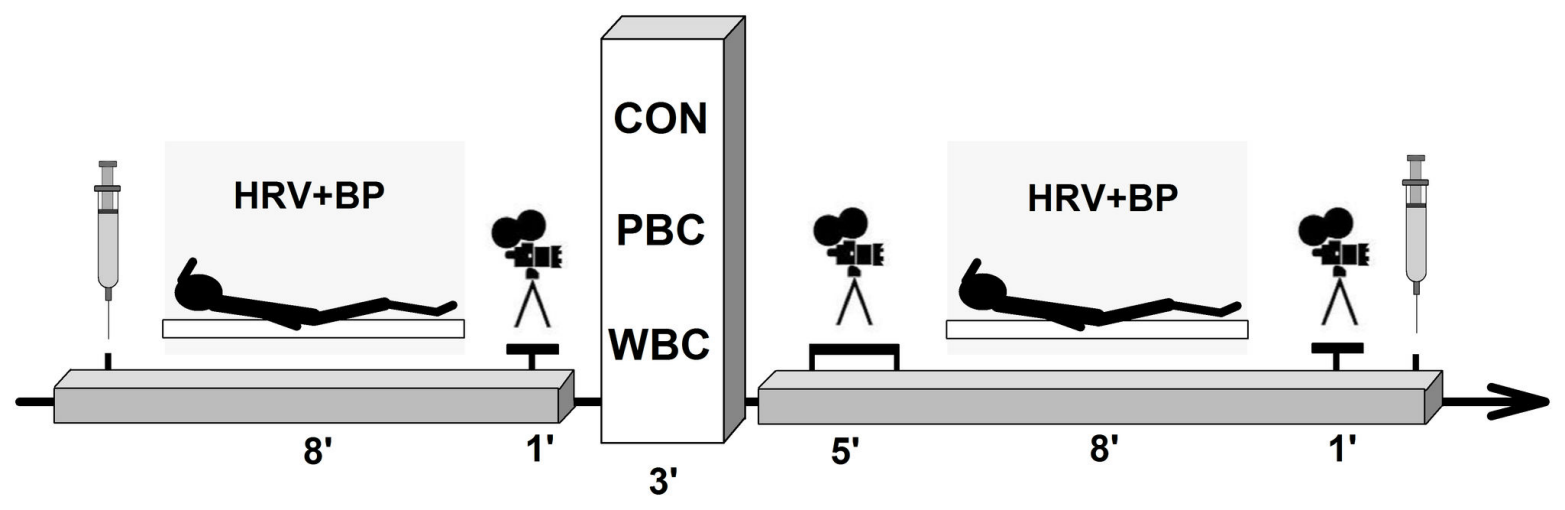
Schematic representation of the experimental protocol. Each subject experienced a unique whole-body cryostimulation (WBC) or partial-body cryostimulation (PBC) session or no session (CON) for 3-min, immediately preceded and followed by the same measurements. Perceived sensations were recorded during each camera recording.

This study was conducted to compare the physiological responses to different cryostimulation techniques on human physiology. One system consisted of a cold room where the subjects were entirely exposed to a very dry and cold air at -110°C, whereas the other system was an open tank exposing the body to -160°C, except the head and neck. The main purpose was to determine whether partial-body cryostimulation (PBC) was as effective as a whole-body cryostimulation (WBC) in stimulating a parasympathetic activation. The secondary purpose was to provide scientific information about a new-generation cryotherapy device (PBC) that is portable and can therefore be used close to the field of training. Hence, 2 groups of 15 subjects performed one WBC or PBC session for 3-min. 10 more subjects composed a control group, without any exposure to cold. Physiological and subjective measurements were performed immediately before and during the 20-min after the exposure (Figure 1).

### Cryostimulation sessions

The investigations were carried out at the medical department of the National Institute of Sport, Exercise and Performance (INSEP, Paris, France), where two different cryotherapy systems were installed. One system consisted of three contiguous WBC chambers maintained at different temperatures; -10°C, -60°C and -110°C (Zimmer Elektromedizin, GmbH, Ulm, Germany), whereas the other system consisted of an open tank where the body was exposed to a very cold temperature (-160°C) except the head and neck (Cryotechno^®^, TEC4H, France). In both systems, the entire cooling process was automatically controlled and the temperatures of exposure remained constant throughout the experiment. The temperature provided by the Zimmer system was the mean ambient temperature recorded inside the cold room, whereas the temperature provided by the Cryotechno^®^ system was that recorded at the entrance of the tank, and not the mean ambient temperature. In both cryotherapy modalities, subjects were exposed to cold for 3-min. The temperature and duration of PBC and WBC were that recommended by manufacturers and were similar to other studies in the literature [9,15,34]. The main differences between the two systems were that whole body was exposed to a cold and dry air in the Zimmer system whereas expanded nitrogen gas was used in the Cryotechno^®^ system, and the head and neck were not exposed to cold. A familiarization session was previously organized with a time exposure reduced to 1-min, and cryostimulation sessions were administered under medical supervision. Moreover, before the cold exposure, the participants were instructed to towel-dry any eventual sweat, wear a bathing suit, surgical mask, earband, triple layer gloves, dry socks and rubber clogs. All jewelry, piercings, glasses and contact lenses were removed before the cold exposure. For the WBC, subjects were instructed by the technician to walk slowly around the chamber, while flexing and extending their elbows throughout the exposure. For the PBC, because of the small space inside the cryotherapy tank, subjects could only slightly turn round and move their wrists slowly.

### Measurements

#### Skin and tympanic temperatures

**Figure 2:**
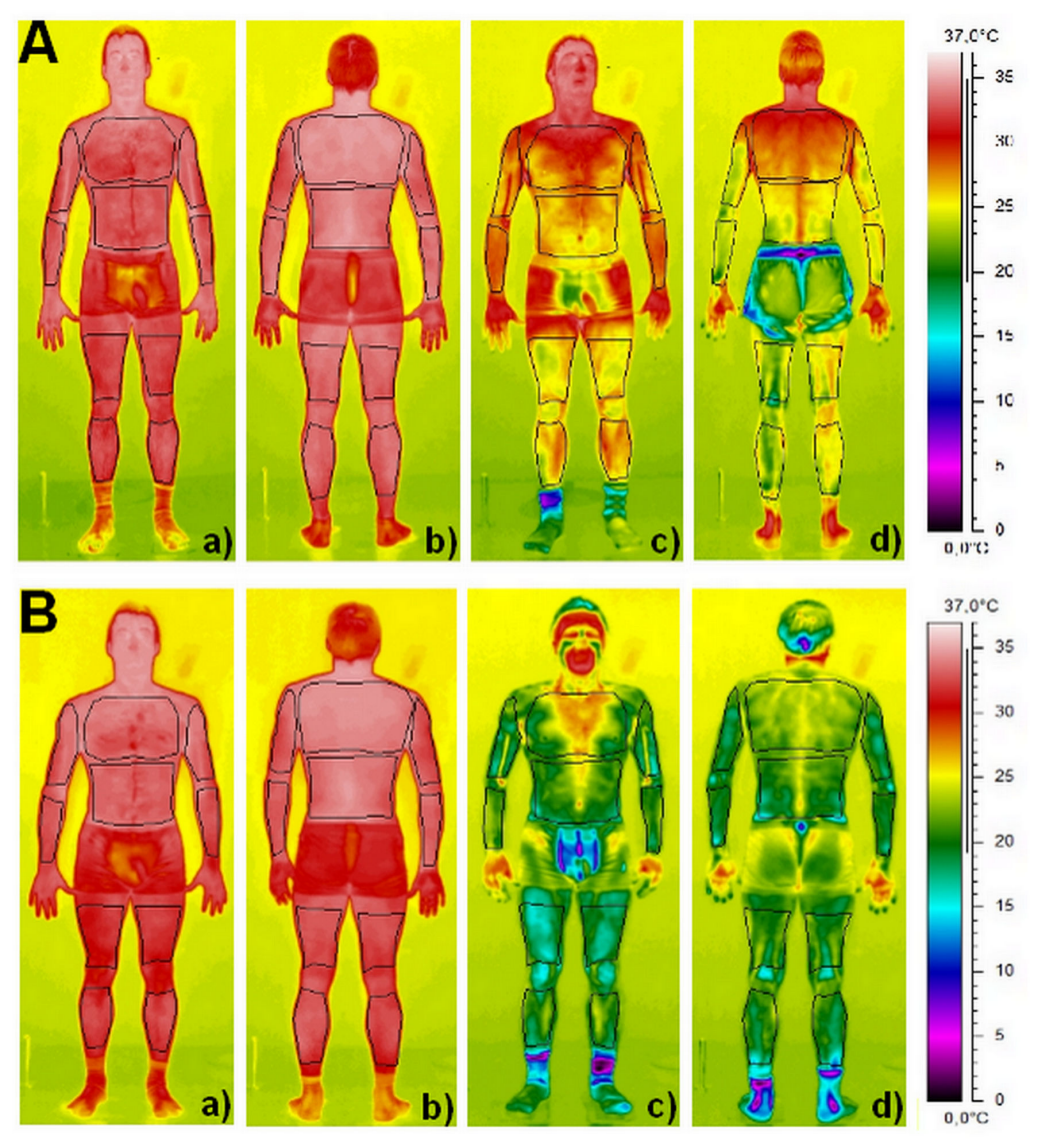
Examples of thermograms obtained immediately before (a, b) and after (c, d) a partial-body cryostimulation (PBC) session (A) and a whole-body cryostimulation (WBC) session (B). The black shapes represent the different body regions of interests for the front and back faces.

Skin temperature was assessed by using a Thermo Vision SC 640 Thermal imaging camera (Flir Systems, Danderyd, Sweden) in accordance with the standard protocol of infrared imaging in medicine [35]. The camera, with the emissivity set in the range of 0.97 to 0.98, was connected to a personal computer with appropriate software (Thermacam Researcher Pro 2.10, Flir systems, Danderyd, Sweden). The camera was mounted on a tripod and positioned in a way to focus on the entire body. The distance between the camera and the subject ranged from 2.5–3.0m (depending on the height and the size of the individual). The thermograms of the chosen body regions of interests were performed before (Pre) and during the first 5-min following the WBC and PBC sessions (Post to P5) and 20-min after (P20) in the temperate room where the temperature was maintained stable (24°C). The subjects were instructed to remain standing in the anatomical position while the thermograms were performed. Participants were also asked to turn round immediately after the exposure to cold (Post), and at the end of each minute (P1 to P5) in order to perform thermograms of the back side. Finally a last front and back thermogram was performed 20-min after the end (P20) of the WBC and PBC exposures. 20 body regions of interests were chosen to study as thoroughly as possible the evolution of skin temperature before and after the cold exposure. 10 regions corresponded to the front side of the body (i.e. torso, abdominal, right and left forearms, right and left arms, right and left thighs, and right and left legs) and 10 regions to the backside (i.e. upper back, lower back, right and left forearms, right and left arms, right and left thighs, and right and left legs) covering almost the whole body (Figure 2). For more clarity, the body regions corresponding to the arms and forearms as well as for legs and thighs, and torso and abdominal, and upper back and lower back, were grouped, giving a mean temperature for arms, legs, and trunk for the front and the back sides. Mean temperature for the whole body was also calculated by averaging the skin temperature recorded for the 20 regions of interests.

Before and after each WBC and PBC session, tympanic temperature (Ttymp) was measured with a tympanic thermometer (Braun Thermoscan^®^ Pro 4000, NY, USA) in order to estimate core temperature. This measurement was performed at Pre, Post, P5, and P20.

#### Blood pressure, heart rate, and HRV indices of parasympathetic activity

Heart rate (HR) was recorded at Pre and P5. For each condition, subjects were comfortably installed in a supine position on a medical bed for 8-min. This test was organized in a dark and quiet room, avoiding any distractions that could induce HR fluctuations. Additionally, the subjects were asked to remain still and not to talk. For all resting HR recordings, R-R intervals were recorded continuously with a Suunto Memory Belt HR monitor with a sampling rate of 1000 Hz, and the capacity to record respiratory rate (Memory Belt, Suunto Oy^®^, Vantaa, Finland).

R–R interval data files were transferred to the computer using the Suunto Training Manager Software and were further analyzed using specialized heart rate variability (HRV) analysis software (Nevrokard^®^ aHRV, Izola, Slovenia). An experienced investigator visually identified and manually removed any occasional ectopic beats and artefacts. Since HRV parameters classically used to study the sympathetic modulation (i.e. SD2 and the low to high frequency ratio) are still a matter of debate, the HRV analysis was restricted to indicators of parasympathetic modulation. The time-varying index kept for analysis was the root-mean square difference of successive normal R-R intervals (RMSSD) [36]. Mean HR was also analyzed. Power spectral density analysis was then performed using a fast Fourier transform with a non-parametric algorithm. The power density of high frequency (HF: 0.15-0.50 Hz) component of the spectrum was calculated to provide an additional index of parasympathetic activity. Both HRV indices of parasympathetic activity were calculated using the last 4-min of the 8-min HR recordings [36]. Moreover, we decided to allow our participants to breathe spontaneously during the measurements [37]. For all HRV samples, it was verified that the respiration rate always remained in the high frequency range (HF: 0.15-0.50 Hz) since the system employed allowed to record this parameter during each test. When this assumption was not met, the test was not retained for subsequent analysis.

Systolic and diastolic blood pressures (Sys BP and Dia BP) were also recorded at the end of the 8-min resting period, by using an oscillometric sphygmomanometer (705 IT, Omron, Kyoto, Japan) positioned on the left arm while the person was in a supine position.

#### Blood analyses

To avoid inter-assay variation, all blood samples were analyzed in a single batch at the end of the study. Before and 30 minutes after the cold exposure, following the imaging, HRV and blood pressure tests, blood samples were collected from a superficial forearm vein using standard venipuncture techniques. 33 ml of blood was directly collected into EDTA tubes for each sample (Greiner Bio-one, Frickenhausen, Germany).

Blood samples were immediately centrifuged at 3000 rev. min^-1^ for 10-min at +4°C to separate plasma from red blood cells. The obtained plasma samples were then stored in multiple aliquots (Eppendorf type, 1500 µL per samples) at -80°C until analysis. A sensitive high-pressure liquid chromatography (Knauer, Berlin, Germany; Column, Lichtopher 60, RP Select B, Merck, Germany) was used for further analysis. Plasma epinephrine, norepinephrine, and dopamine were determined by means of an electrochemical detector (2143-RPE, Pharmacia LKB, Freiburg, Germany) and computed as ng.L^-1^.

#### Thermal and comfort sensations

The thermal and comfort sensations of participants were recorded at Pre, Post, P5 and P20 using scales of perceived thermal and comfort [38]. The subjects rated their thermal sensation with a nine-point standard scale before and after WBC and PBC by answering the question ‘How are you feeling now?’. They were instructed to answer by instinctively giving a number ranging from 4 to -4 (4 = very hot, 3 = hot, 2 = warm, 1 = slightly warm, 0 = neutral, -1 = slightly cool, -2 = cool, -3 = cold, -4 = very cold). Thermal comfort was also rated with a five-point scale by answering the question ‘How do you find this?’. Participants were instructed to answer by instinctively giving a number ranging from 0 to 4 (0 = comfortable, 1 = slightly uncomfortable, 2 = uncomfortable, 3 = very uncomfortable, 4 = extremely uncomfortable).

In addition, the air temperature and the relative humidity of the rooms used for measurements were recorded so that it was possible to control the confounding effects of potential changes in the environment.

### Statistical analysis

We tested the normality of each variable from a normal probability plot and by using the Shapiro-Wilk test. These analyses were performed using Statistica software (7.0 version, Statsoft, France). Because the cardiovascular and blood parameters data did not always meet the assumptions of normality, they were log-transformed to reduce bias arising from non-uniformity error. Data were then analyzed for practical significance using magnitude-based inferences [39]. All qualitative analyses were conducted using a modified statistical spreadsheet [40]. We used this qualitative approach because traditional statistical approaches often do not indicate the magnitude of an effect, which is typically more relevant for clinical or practical prescription than any significant effect. Between-trial standardized differences or effect sizes [ES, 90% confidence limits (CL)] in cardiovascular parameters, catecholamine concentrations, skin and core temperatures, and perceived sensations, were calculated by using the pooled standard deviation [41]. Threshold values for Cohen ES statistics were ≤ 0.2 (trivial), > 0.2 (small), > 0.5 (moderate), and > 0.8 (large). Quantitative chances of higher or lower differences were evaluated qualitatively as follows: <1%, *almost certainly not*; 1-5%, *very unlikely*; 5-25%, *unlikely*; 25-75%, *possible*; 75-95%, *likely*; 95-99%, *very likely*; >99%, *almost certain*. If the chance of higher or lower differences was >5%, the true difference was assessed as unclear. Otherwise, we interpreted that change as the observed chance [39]. Data in text and figures are presented as mean±90% CL.

## Results

### Skin and tympanic temperatures

**Figure 3:**
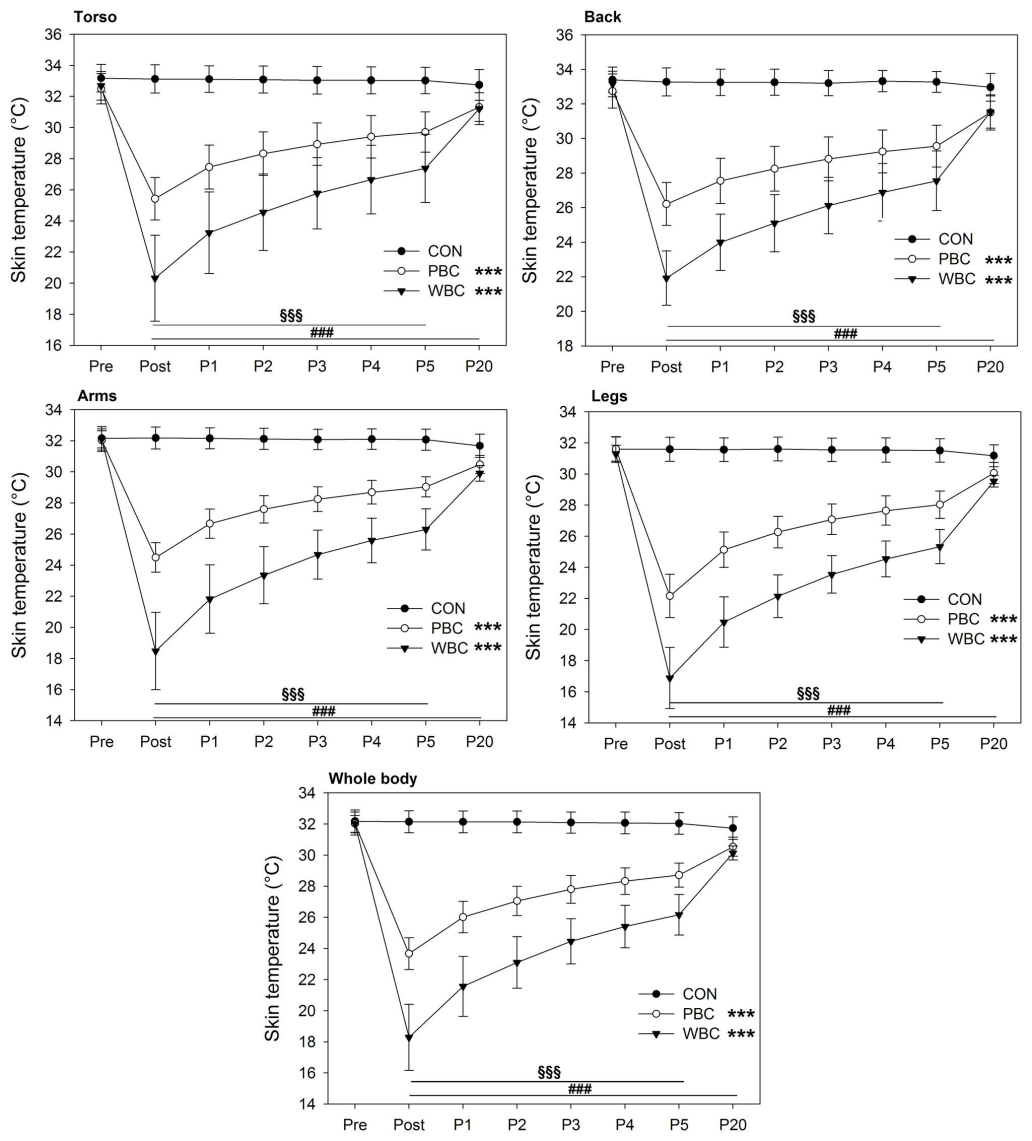
Changes in the mean skin temperature of the different body regions of interests. Values were recorded before (Pre), immediately after (Post) and for 20-min (P1 to P20) after whole-body cryostimulation (WBC), and partial-body cryostimulation (PBC) sessions, and in the control (CON) condition. Within-group change (Post conditions vs. Pre): * likely; ** very likely; *** almost certain. Between-group (vs. CON) difference in the change: # likely; ## very likely; ### almost certain. Between-group (PBC vs. WBC) difference in the change: § likely; §§ very likely; §§§ almost certain.

Baseline skin temperature (Tskin) for all body regions was similar between groups before the cryostimulation (Figure 3). For all body regions, there was an *almost certain* very large reduction in mean skin temperature within the 20-min after the WBC and PBC exposures (Figure 3). The mean decrease in Tskin for the whole body was -8.3°±0.3°C (chances that the true difference was lower/trivial/higher, 100/0/0%, ES±90% CL, -8.1±0.4) immediately after (Post) the PBC session, and -13.7±0.7°C (100/0/0%, ES, -6.2±0.5) after the WBC session. The change in Tskin for all body regions was *almost certainly* greater in the WBC group than in the PBC group at Post and P5 and *almost certainly* greater in the WBC and PBC groups than in the CON group up to P20 (Figure 3).

**Figure 4:**
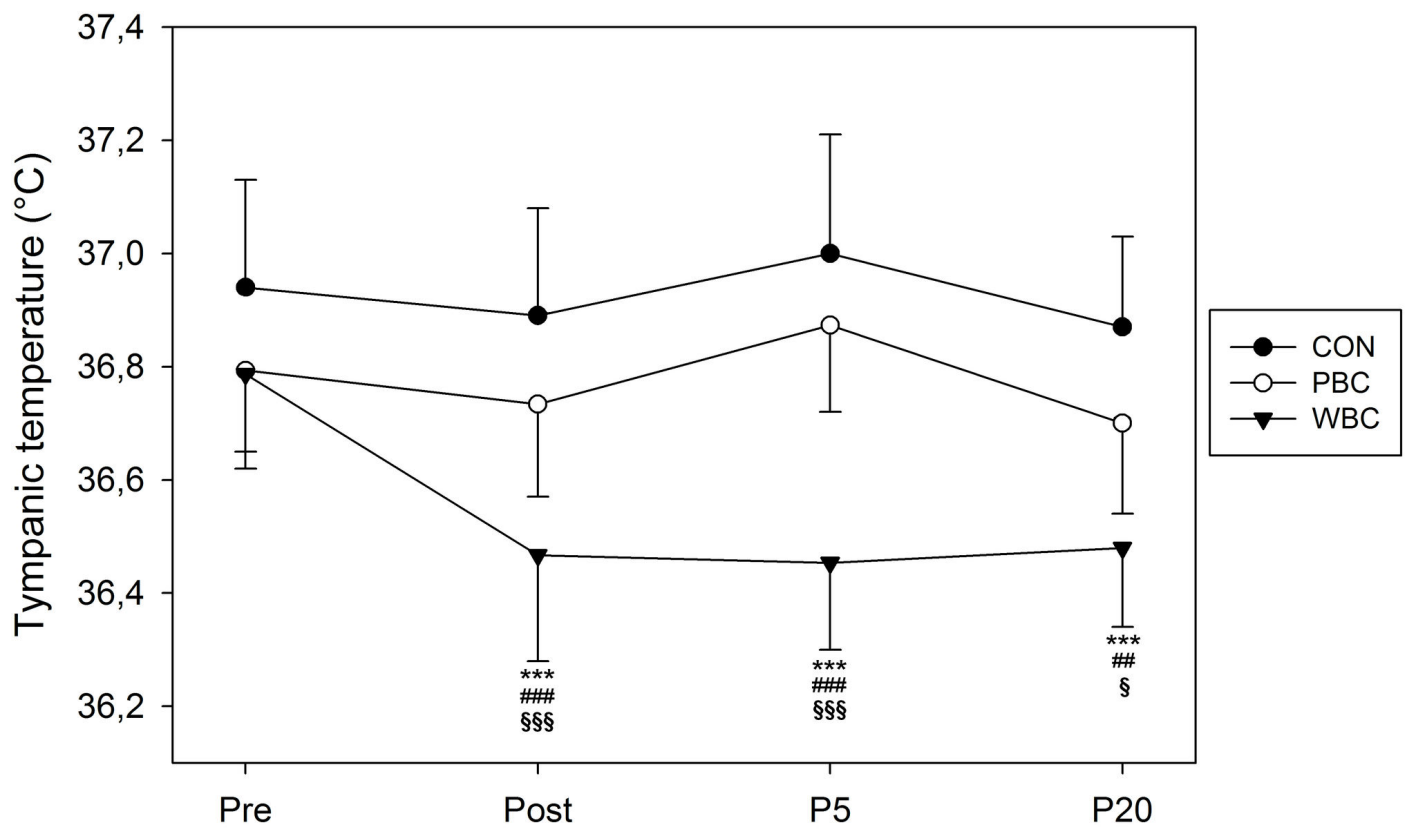
Changes in tympanic temperature. Values were recorded before (Pre), immediately (Post), 5-min (P5), and 20-min (P20) after whole-body cryostimulation (WBC), and partial-body cryostimulation (PBC) sessions, and the control (CON) condition. Within-group change (Post conditions vs. Pre): * likely; ** very likely; *** almost certain. Between-group (vs. CON) difference in the change: # likely; ## very likely; ### almost certain. Between-group (PBC vs. WBC) difference in the change: § likely; §§ very likely; §§§ almost certain.

In addition, the tympanic temperature (Ttymp) *almost certainly* declined only after the WBC session (within-group change±90% CL, -0.32±0.04°C, chances that the true difference was lower/trivial/higher, 100/0/0%, ES±90% CL, -0.85±0.20), and *almost certainly* remained lower than baseline values up to 20-min after the exposure (-0.30±0.11°C, 100/0/0%, ES, -0.82±0.29). Changes in Ttymp were *trivial* in PBC and CON groups (Figure 4).

### Cardiovascular parameters

**Figure 5:**
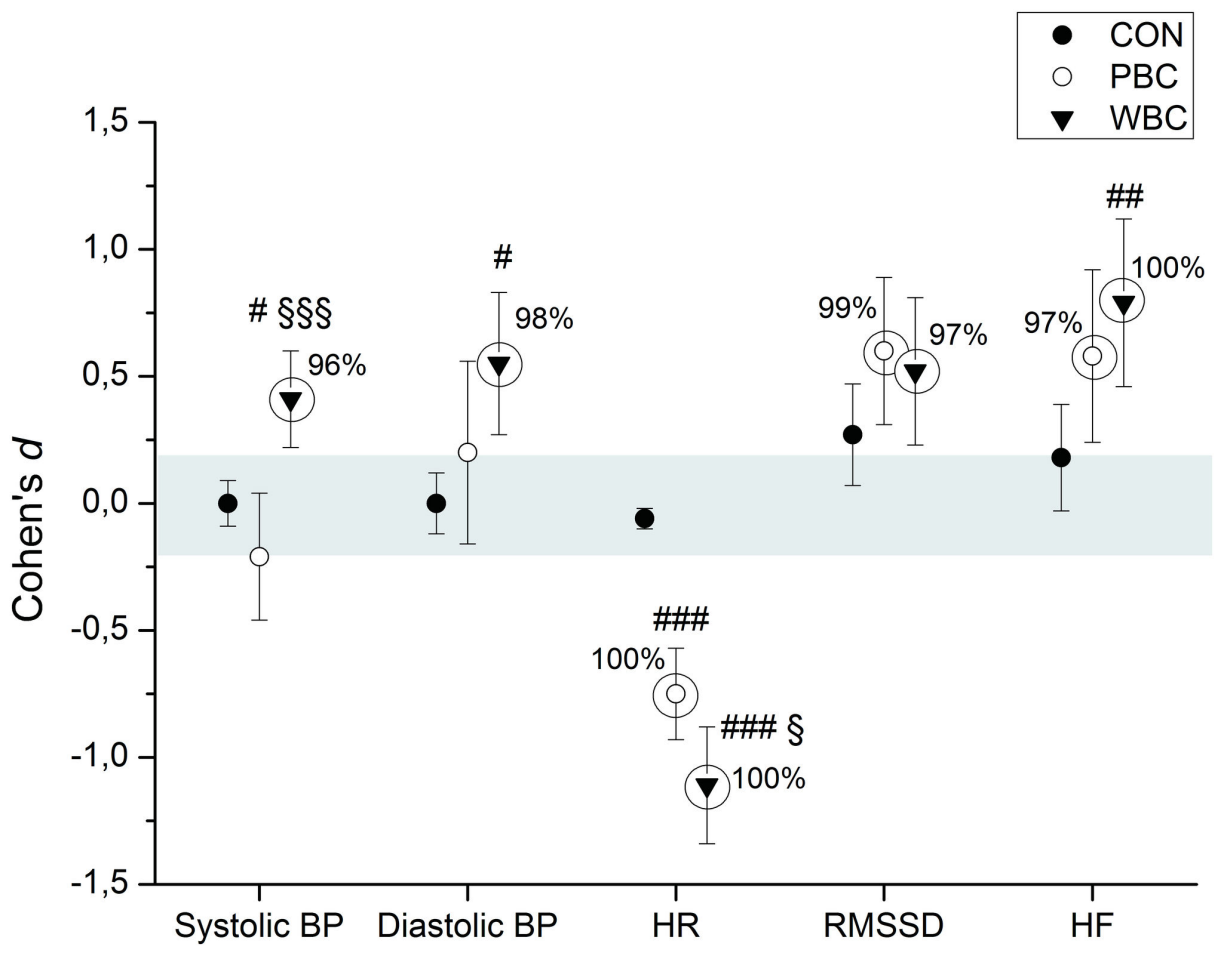
Changes (Cohen’s *d* or effect size) in blood pressure, heart rate and HRV indices of parasympathetic activity from pre to post whole-body cryostimulation (WBC), and partial-body cryostimulation (PBC) sessions, and for the control (CON) condition. Circles around the plots highlight *very likely* to *almost certain* differences in the change. The shaded area represents the smallest worthwhile change. Between-group (vs. CON) difference in the change: # likely; ## very likely; ### almost certain. Between-group (PBC vs. WBC) difference in the change: § likely; §§ very likely; §§§ almost certain.

Systolic and diastolic BP were *very likely* increased after the cryostimulation in the WBC group (0/4/96%, ES, +0.41±0.19, and 0/2/98%, ES, +0.55±0.27, for Sys BP and Dia BP, respectively), whereas changes were *trivial* in the PBC and CON groups. In addition, HR *almost certainly* decreased by -6.8±1.8 bpm (100/0/0%, ES, -0.75±0.18) after the PBC session, and by -9.4±2.1 bpm (100/0/0%, ES, -1.11±0.23) after the WBC session. HR changes were *almost certainly* different from the CON group, and the change recorded after the WBC session was *likely* greater than after the PBC session (Figure 5). RMSSD was *very likely* increased after the PBC (0/1/99%, ES, +0.60±0.29) and WBC sessions (0/3/97%, ES, +0.52±0.29), while HF was *very likely* increased after the PBC (0/3/97%, ES, +0.58±0.34), and *almost certainly* increased after the WBC (0/0/100%, ES, +0.79±0.33) (Figure 5). The increase in HF after WBC was *very likely* greater than the CON group (0/2/98%, ES, +0.66±0.38), and *likely* greater than the PBC group (1/12/88%, ES, +0.52±0.47), but in moderate proportions (ES<0.8).

### Plasma catecholamine concentrations

**Figure 6:**
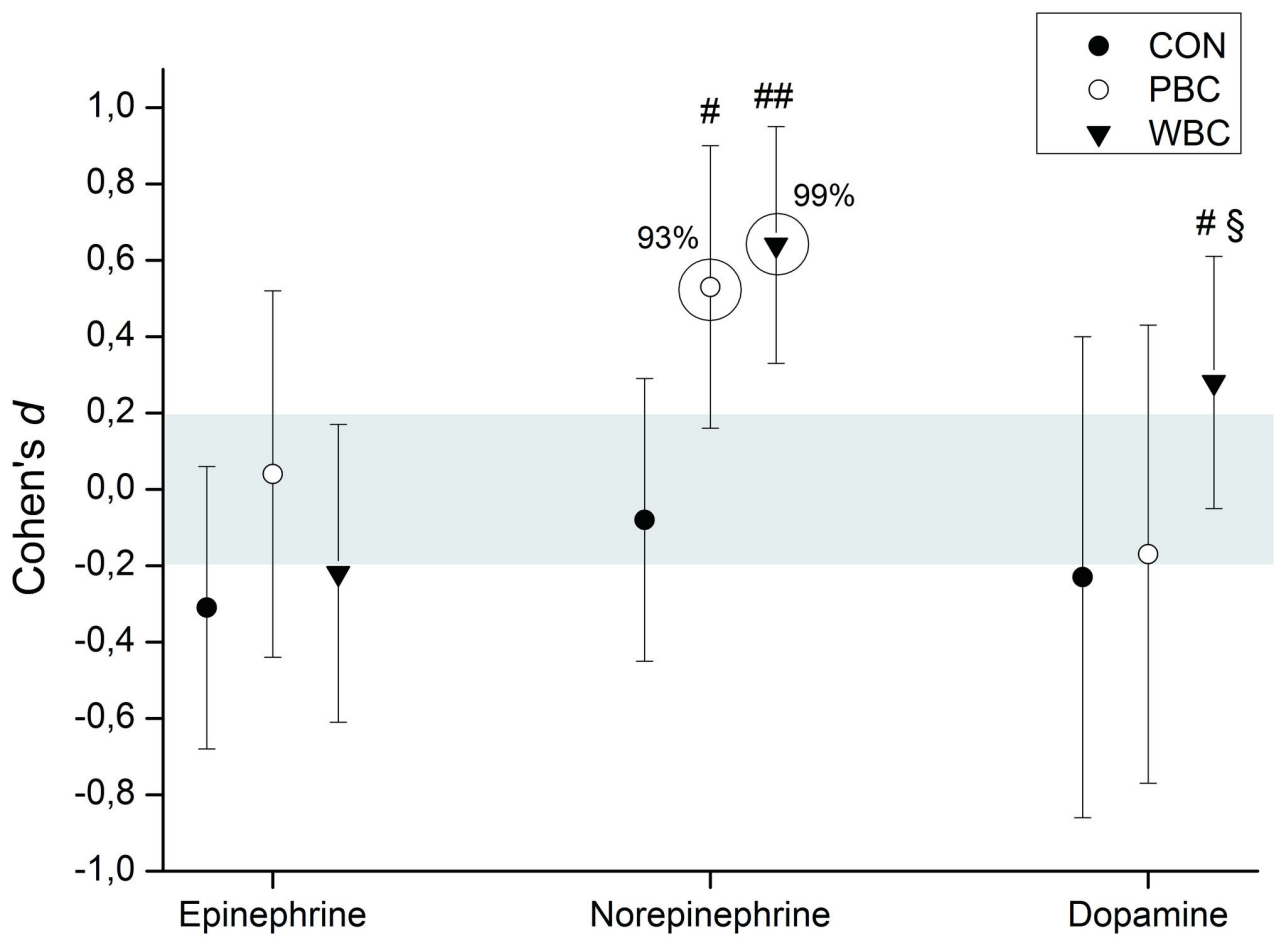
Changes (Cohen’s *d* or effect size) in plasma concentrations in catecholamines from pre to post whole-body cryostimulation (WBC), and partial-body cryostimulation (PBC) sessions, and for the control (CON) condition. Circles around the plots highlight *likely* to *almost certain* differences in the change. The shaded area represents the smallest worthwhile change. Between-group (vs. CON) difference in the change: # likely; ## very likely; ### almost certain. Between-group (PBC vs. WBC) difference in the change: § likely; §§ very likely; §§§ almost certain.

Cold-induced changes in plasma catecholamine concentrations are depicted in Figure 6. The PBC session *likely* induced a *moderate* increase (0/7/93%, ES, +0.53±0.36) in plasma norepinephrine concentration, while this increase was *very likely* after the WBC session (0/1/99%, ES, +0.64±0.31). These changes in plasma norepinephrine concentrations obtained after PBC and WBC sessions were respectively *likely* and *very likely* greater than that obtained in the CON condition (Figure 6). Within-group changes in plasma epinephrine and dopamine concentrations were trivial in PBC and CON groups. Only WBC *possibly* induced a small increase (1/32/67%, ES, +0.28±0.33) in plasma dopamine concentration and this change was *likely* greater than after PBC (5/20/75%, ES, +0.46±0.64) and CON conditions (4/17/79%, ES, +0.53±0.70).

### Thermal and comfort sensations

**Figure 7:**
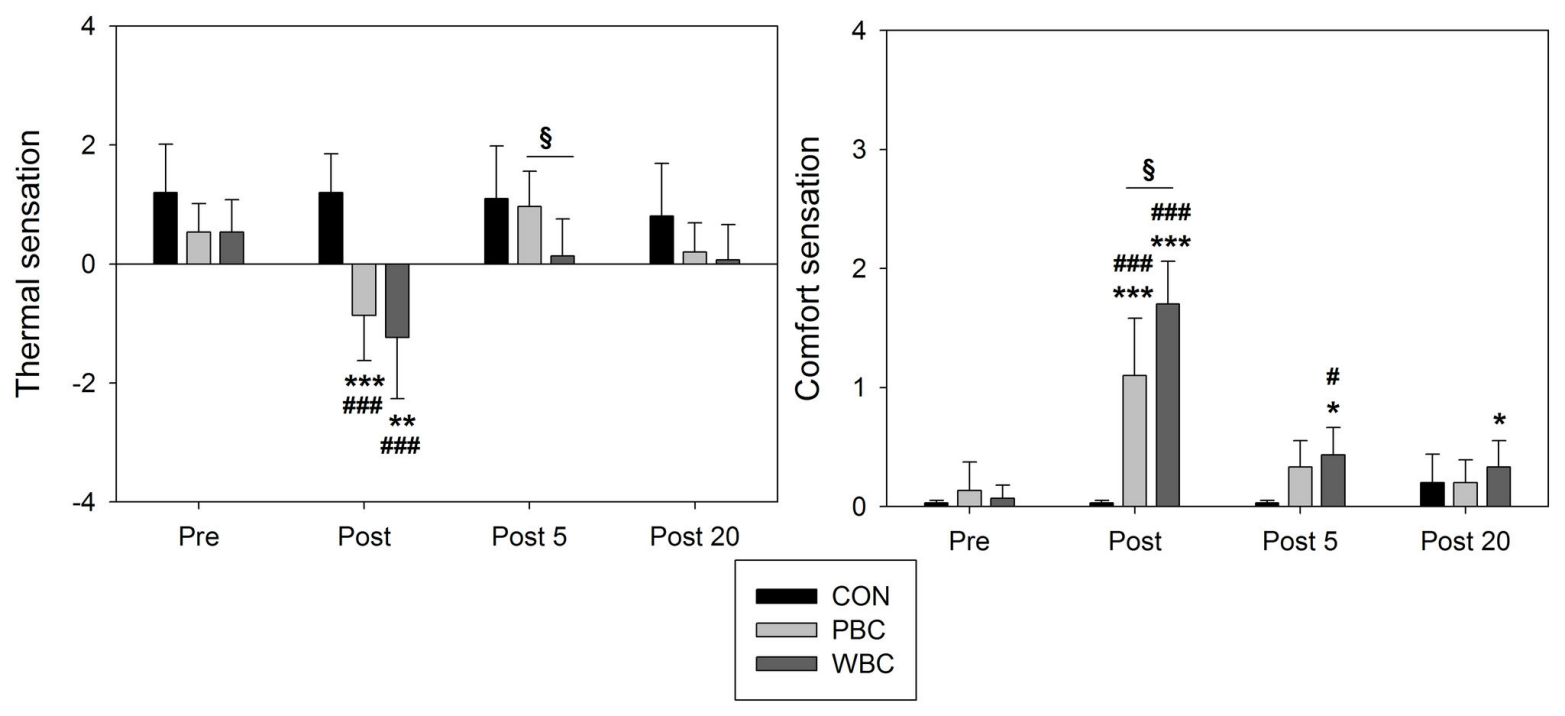
Changes in thermal and comfort sensation scores. Values were recorded before (Pre), immediately (Post), 5-min (P5), and 20-min (P20) after whole-body cryostimulation (WBC), and partial-body cryostimulation (PBC) sessions, and the control (CON) condition. Within-group change (Post conditions vs. Pre): * likely; ** very likely; *** almost certain. Between-group (vs. CON) difference in the change: # likely; ## very likely; ### almost certain. Between-group (PBC vs. WBC) difference in the change: § likely; §§ very likely; §§§ almost certain.

Both PBC and WBC modalities induced an *almost certain* alteration in thermal and comfort sensations immediately after (post) the exposure (Figure 7). This alteration was *almost certainly* different than in the CON condition. Subjects perceived the WBC exposure as *likely* more uncomfortable than the PBC (1/7/93%, ES, +0.87±0.74). This sensation of discomfort compared with baseline values, *likely* persisted up to 20-min after the WBC exposure (0/12/87%, ES, +0.43±0.33).

## Discussion

Considering the popularity of cryotherapy in sports medicine, the present study compared for the first time the potential effects of WBC and PBC on autonomic nervous system activity. Whatever the cryotherapy technique used, results showed that a single 3-min cryostimulation induced a strong autonomic response, as rising plasma norepinephrine, systolic and diastolic blood pressures reflected increased sympathetic activation, and as the rise in HRV indices suggested an augmentation of the parasympathetic control of heart rate. A *likely* greater parasympathetic activation was observed with the greatest body cooling obtained by exposing the whole body (WBC) to cold.

### Thermal and subjective responses to cryostimulation

Two main modalities of air-based cryotherapy are currently employed by physiotherapists and physicians for sport recovery, injury and rehabilitative purposes, and as an alternative treatment for inflammatory pathologies and anxiety-depression disorders [6,7,10]. Contrarily to localized cryotherapy obtained by the application of ice packs, cold towels, or cold air-pulsed on a small body region, the air-based cryotherapy modalities examined in the present study involved either complete body cooling (WBC) or whole-body cooling except the head and neck (PBC), inducing an important decrease in whole-body temperature. An *almost certain* very large reduction in Tskin of all body regions of interests was recorded after the 3-min cryostimulation, in greater proportion with the WBC. The mean decrease in Tskin for WBC was of 13.7°C (i.e. -42.9%) and 8.3°C (i.e. -26.1%) for PBC. As previously reported by Cholewka et al. (2012), a larger decrease in Tskin was recorded in the legs and arms when compared with the torso and back (Figure 2). These authors reported a significant positive correlation between the decrease in Tskin and the BMI of individuals, indicating that the effects of cryostimulation may be influenced by body composition. Accordingly, body composition was an important selection criterion for the participants in the present study. Recently, Savic et al. [42] studied the air temperature inside an open cryotherapy tank providing liquid nitrogen (-140°C to -195°C), and showed that the lower part of the cryotherapy tank was colder than the upper part, which could also explain the differences in cooling between body regions. Cold air has higher density than warm air, which means lower parts of the cryotherapy tank are colder than upper. Although previous studies have reported a similar decrease in Tskin as ours after a WBC session, the present study is one of the first to report the cooling effect of PBC [42,43]. Even though this recent cryotherapy technique based on expanded nitrogen gas provided a colder temperature (-160°C) compared to WBC (110°C), it did not induce the greatest body cooling, due to the opening of the cryotherapy tank to ambient temperature. The thermograms presented in the recent study of Savic et al. [42] confirm the difference in body cooling obtained in ours between WBC and PBC. As previously shown by Costello et al. [43] with a cryochamber and by Savic et al. [42] with an open cryotherapy tank, whatever the cryotherapy modality, Tskin remained lower than basal values 20-min after the exposure to cold, with similar recovery patterns in both modalities. However, this decrease in Tskin was associated with an *almost certain* reduction in Ttymp, persisting up to 20-min after the exposure only with WBC, suggesting potentially greater physiological modifications when WBC is used. Similarly, Costello et al. [44] reported a significant ~0.3°C reduction in Ttymp after a 4-min WBC session, without comparison with a control group. However, there is still no consensus regarding ideal reductions in skin or tympanic temperatures, with the exception of one study reporting that a skin temperature below 12°C would be required to observe a 10% decrease in nerve conduction velocity, inducing the analgesic effect sought by patients with inflammatory pathologies [45]. Within this context, the present study may provide new insight about whether a larger reduction in body temperature may exert beneficial effects on the activity of the autonomic nervous system (ANS). In addition, both cryotherapy modalities induced an alteration of perceived sensations of cold and comfort. Immediately after the exposure to cold, subjects felt “slightly cool to cool” (between -1 and -2) without differences between cryotherapy groups, while WBC (1.7±0.3; indicating “uncomfortable”) was *likely* perceived as more “uncomfortable” than PBC (1.1±0.5; indicating “slightly uncomfortable”). A very slight “uncomfortable” sensation (0.3±0.2) *likely* persisted up to 20-min after the WBC session. These data suggest that, despite extreme cold temperatures, both modalities of cryotherapy were well tolerated by subjects not previously accustomed to cryostimulation. However, it is important to take into account that the present study was carried out in the winter season, and thus the participants were probably already somewhat habituated to cold weather. Greater differences from baseline values in the changes in thermal and comfort sensations would be probably obtained if the study was conducted during the summer [38].

### Cold-related stimulation of the autonomic nervous system

HRV indices and blood catecholamines are classically used to evaluate the modulation of the ANS in response to various stimuli such as cold or physical exercise [12,20,46]. The parasympathetic and sympathetic activities refer to the cholinergic and adrenergic phases of the ANS, in reference to their respective neurotransmitters (i.e. acetylcholine for the parasympathetic component, and catecholamines for the sympathetic component). Given the lack of consensus on the accuracy of HRV analysis in assessing sympathetic activity, in the present study the activity of this component of the ANS was studied only through plasma catecholamine concentrations, while the parasympathetic component was studied through HRV analyses. Plasma noreprinephrine concentrations were *likely* and *very likely* increased after the PBC and WBC sessions, respectively, suggesting increased sympathetic nerve stimulation. This increase in plasma norepinephrine was accompanied by a *possible* small increase in plasma dopamine after WBC only, but no response in plasma epinephrine was recorded after PBC and WBC sessions. Similar findings, cold-induced increases in plasma norepinephrine without any changes in plasma epinephrine, have been reported after different modalities of cold exposure [47,48]. Since norepinephrine mostly originates from the sympathetic nerve endings and epinephrine from the adrenal medulla, we can suggest that both cryostimulation techniques activate the sympathetic nerve system. In addition, since an increase in plasma dopamine is typically related to sensations of well-being and pleasure, we can suggest a slightly greater effect of WBC in generating positive feelings. A previous study reported a significant increase in sensations of well-being when an exhaustive treadmill running protocol was followed by a WBC session [9].

During cryostimulation, cold-sensitive cutaneous receptors excite the sympathetic α-adrenergic fibres, responsible for a peripheral vasoconstriction mechanism through the release of norepinephrine. Consequently, blood flow is redistributed toward the core, resulting in increased arterial pressure [34]. In the present study, Sys BP and Dia BP were *very likely* increased after WBC, but not after PBC, pointing to a lower sympathetic stimulation that may be related to the smaller decrease in Tskin obtained after PBC. Further, the decrease in Ttymp recorded with WBC, as well as the stimulation of cold trigemino-cardiac reflex receptors located in the face may have accentuated the parasympathetic response after WBC, augmenting vagal output to the heart. As expected, the increase in BP was associated with a large decrease in HR, that was larger after WBC (-15.2%) than PBC (-10.9%) likely reinforced by the concomitant triggering of the baroreflex which lowers the sympathetic tone of the ANS, shifting to a predominance of the parasympathetic tone [25].

HRV indices of parasympathetic activity (RMSSD and HF) were *very likely* to *almost certainly* increased after the cryostimulation without marked differences between WBC (RMSSD: +85.2%; HF: +632%) and PBC (RMSSD: +54.4%; HF: +138%) (Figure 5). Increases in HRV indices of parasympathetic activity were previously observed after a single WBC session at -110°C in non-athletic women (RMSSD: +53%) [24] and in elite synchronized swimmers (RMSSD: +78%; HF: +296%) [12]. Cold water immersion (5-min in 11 to 14°C) was also shown to increase RMSSD and HF values but in lower proportions compared to WBC [18–20]. According to these findings, a larger thermal stress obtained with WBC would induce a greater stimulation of cardiac parasympathetic activity than immersion or PBC. This greater effect of WBC could be reinforced by the greater magnitude of the baroreflex concomitantly to the activation of trigemino-cardiac reflex receptors when the face is exposed to cold. Al Haddad et al. [20] recorded a 26.2% greater increase in RMSSD value after a 5-min cold water face immersion compared to a control condition, after a submaximal exercise. While RMSSD did not differ between WBC and PBC in the present study, the increase in HF was *very likely* (0/2/98%) but moderately greater after WBC than PBC, with a moderate effect size (ES, 0.52±0.47). One explanation might be greater movements and greater changes in respiratory patterns during the WBC session, compared to PBC, which could have slightly increased the sympathetic activity and/or lowered the parasympathetic activity. Moreover, it can be presumed that the parasympathetic activation had reached its plateau following PBC, and it might not be useful to induce a larger thermal stress to obtain the desired physiological responses. Indeed, a saturation of cardiac parasympathetic regulation has been previously reported, in conditions involving an already heightened vagal tone, such as in highly trained individuals [49,50]. Indeed, although an increase in training load has been found to enhance cardiac parasympathetic activity in moderately trained individuals, it had no effects in already highly trained individuals [49]. The main explanations for a saturation of parasympathetic activity may include a loss of phasic vagal efferent discharge at high level of vagal activity (as a result of high neuronal vagal excitability), or saturation of acetylcholine receptors [51,52]. Buch et al. [53] even reported a fall in HRV indices of parasympathetic activity in highly-trained individuals in response to an overload training period. These results question the optimal temperature of cryostimulation (i.e. the parasympathetic stimulus), and suggest that the magnitude of the parasympathetic response following a cryostimulation could be dependent of the initial level of the parasympathetic activity. Within this context, WBC would be recommended as an acute treatment when a large parasympathetic reactivation is needed such as in individuals suffering from burn-out, depressive symptoms, or suffering from a poor sleep quality, while PBC would be useful as a chronic treatment to maintain a high parasympathetic tone, for example when physical exercise bouts are repeated such as during a cycling stage race or a tennis tournament.

### Implications for the clinical use of different techniques of cryotherapy

Considering the widespread use of cryotherapy in medicine and in athletic recovery, a first assessment and comparison of the physiogical effects of the two main dry-air techniques available was needed. The results of this study support the use of cryotherapy, particularly when parasympathetic stimulation is sought. Elevated parasympathetic activity at rest is classically associated with health and well-being, and is stimulated by regular physical activity [49,50]. In the immediate post-exercise period, an increase in the parasympathetic modulation of heart rate is also a crucial physiological reaction necessary to initiate cardiodeceleration and a complete recovery [54]. Conversely, a delayed or incomplete parasympathetic reactivation after exercise is associated with impaired recovery, as well as with an increased risk of ventricular fibrillation and sudden cardiac death [55]. Thus, parasympathetic activity is thought to afford a cardioprotective background, and is probably a valid indicator of post-exercise recovery in athletes. As expected, in the present study, both cryotherapy techniques induced a parasympathetic stimulation in subjects at rest. This reinforces the interest of using cryostimulation in situations requiring a parasympathetic stimulation such as the post-exercise recovery period, especially when the exercise is performed in the heat [56], or between two maximal exercise bouts [12]. HR *almost certainly* decreased and HRV indices of parasympathetic activity *almost certainly* increased after only a single 3-min cryostimulation of WBC and PBC. As inferred through HR values, the parasympathetic stimulation tended to be more pronounced with WBC than PBC, likely related to the greater cooling effect of WBC and/or its specific effects on face. Indeed, the concomitant greater cooling effect and the exposure of the face to cold in WBC may have accentuated the parasympathetic reactivation by increasing respectively, the magnitude of the baroreflex and stimulating the trigemino-cardiac reflex receptors located in the face. In another hand, the increases in the HRV indices of parasympathetic activity were not clearly different between WBC and PBC, still questioning on the optimal procedure of cryostimulation. Further research manipulating the temperature of cryostimulation and isolating the independent effects of cold intensity and face exposure to cold are warranted. Moreover, although a greater degree of cooling and/or face exposure to cold seem to induce larger effects, we do not know how long the effects of WBC on parasympathetic modulation may last, and whether cumulative effects could be obtained after successive WBC sessions. Conversely, individuals accustomed to cryostimulation might progressively derive less benefit from the sessions. Finally, both WBC and PBC induced a large stimulation of the ANS, with predominance of parasympathetic tone, and in greater proportion with WBC. According to these results, WBC should be preferentially used as an acute treatment when the parasympathetic activity is dramatically suppressed. However, PBC would be more indicated as a routine technique that athletes can directly use in the field between two training sessions or competitions, in order to speed-up recovery.

## Conclusion

The present study demonstrates that a single WBC (3-min at -110°C in a cryochamber) or PBC (3-min at -160°C in an open tank) session induces an immediate stimulation of the ANS with a predominance of the parasympathetic tone, as inferred from heart rate and heart rate variability indices. Although both cryotherapy techniques reduced Tskin from baseline, WBC elicited a greater decrease compared to PBC and a significant decrease in Ttymp, which could explain the more pronounced stimulation of the ANS recorded after a WBC session. These data suggest the more the body is cooled, the more the ANS is stimulated, with a larger effect on the parasympathetic tone. However given logistical demands of WBC, PBC may be an appropriate and transportable technique to use in the field.
